# The Cytotoxic and Inhibitory Effects of Plant Derivatives on *Candida albicans* Biofilms: A Scoping Review

**DOI:** 10.3390/molecules28010130

**Published:** 2022-12-23

**Authors:** Manuela Loaiza-Oliva, Laura Arias-Durango, María Cecilia Martínez-Pabón

**Affiliations:** Laboratory of Oral Microbiology, Faculty of Dentistry, Universidad de Antioquia, Medellín 050010, Colombia

**Keywords:** biofilms, *Candida albicans*, plant extracts, toxicity

## Abstract

*Candida albicans* infections are related to biofilm formation. The increase in antifungal resistance and their adverse effects have led to the search for therapeutic options as plant derivatives. This scoping review aims to identify the current status of in vitro research on the cytotoxicity and inhibitory effects of plant derivatives on *C. albicans* biofilms. In this study, PRISMA items were followed. After recognition of the inclusion criteria, full texts were read and disagreements were resolved with a third party. A risk of bias assessment was performed, and information was summarized using Microsoft Office Excel. Thirty-nine papers fulfilling the selection criteria were included. The risk of bias analysis identified most of the studies as low risk. Studies evaluated plant derivatives such as extracts, essential oils, terpenes, alkaloids, flavonoids and polyphenols. Some studies evaluated the inhibition of *C. albicans* biofilm formation, inhibition on preformed biofilms or both. The derivatives at concentrations greater than or equal to those that have an inhibitory effect on *C. albicans* biofilms, without showing cytotoxicity, include magnoflorin, ellagic acid, myricetin and eucarobustol from *Eucalyptus robusta* and, as the works in which these derivatives were studied are of good quality, it is desirable to carry out study in other experimental phases, with methodologies that generate comparable information.

## 1. Introduction

*Candida albicans* is an opportunistic fungus that can cause superficial and systemic infections in individuals when mucosal barriers are disrupted, or when the immune system is compromised [1]. Some factors associated with candidemia in intensive care units (ICU) are long durations of central venous catheterization, urinary tract catheterization and mechanical ventilation [2,3]. At low levels, *Candida albicans* colonizes oral mucosal surfaces [4] as a normal inhabitant, but, under certain circumstances, it can cause a superficial candidiasis characterized by the appearance of white plaques on inflamed and red mucosa and by pain when eating or swallowing [4,5]. If *C. albicans* infection becomes invasive, it can cause septicemia [6]. *C. albicans* has also been reported to infect oral mucositis lesions [7,8], causing inflammation of the oropharyngeal mucosa [9,10]. Patients who suffer from cancer affecting the head and neck, and who receive chemotherapy and radiotherapy treatments, are almost all affected by oral mucositis [9,10].

*C. albicans* infections are related to several virulence factors, including biofilm formation (BF) on implanted medical devices and oral mucosa (biotic and abiotic surfaces) [4,5,6], which allow the initial adhesion to epithelial cells with subsequent tissue invasion, damage and antimicrobial resistance increased risks [5,11,12]. Further, *C. albicans* virulence factors include its ability to switch from the yeast form to an invasive hyphae morphotype, and to secrete proteolytic and lipolytic enzymes [13].

Given its ability to grow in biofilms, *C. albicans* can tolerate higher concentrations of antimicrobials, which has an important impact on public health [14], and has led to an increase in research on drug resistance [15] and therapeutic options. Essential oils, extracts and other plant derivatives are among the most evaluated alternatives, being an attractive option as they provide the possibility of achieving better therapeutic effects with less toxicity and due to their multiple possibilities of use: systemic, topical and as antiseptic in abiotic surfaces.

On the other hand, variations in the techniques for studying cytotoxicity and antifungal capacity make it difficult to identify, in the literature, the plant derivatives with the best level of progress and scientific evidence that supports a possible application.

In this scoping review, we provide an overview of the antifungal *C. albicans* biofilms and the cytotoxic effect of plant derivatives, such as essential oils, extracts and terpenes, placing a particular emphasis on the in vitro information with good quality, the most common used technics and evaluation protocols.

## 2. Results

### 2.1. Essential Oils and Composition

**Figure 1:**
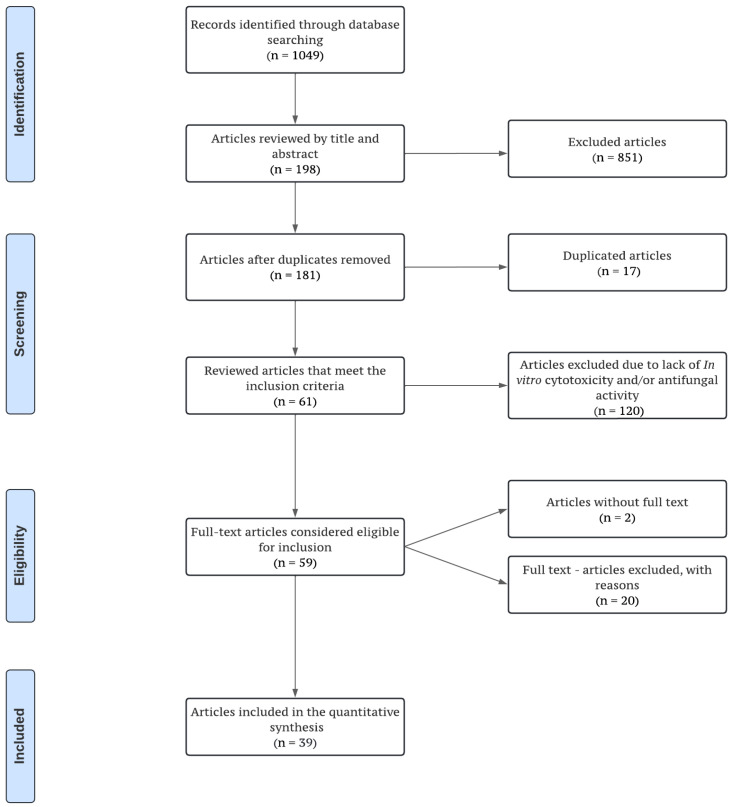
Screening and selection process flow chart, according to the PRISMA statement.

The flow diagram of the screened manuscripts (Figure 1) shows a total of 1049 potentially eligible studies following the electronic screening strategy search. Reviewer agreement led to the elimination of 851 articles that did not conduct an evaluation of the effect of plant derivatives on *C. albicans* biofilms. After removing duplicates, 181 articles were reviewed, resulting in 120 non-eligible studies being excluded at this stage for not having antifungal evaluation at the planktonic level or cytotoxicity assays. Two articles without access to the full text were removed and, finally, fifty-nine full-text articles were reviewed, out of which thirty-nine studies fulfilled all of the selection criteria and were included in the full data analysis. All the included studies were published over a twelve-year period (from 2010 to 2022), with the period from 2014 to 2017 having the highest number of publications (46%).

Publications from five continents were identified; Brazil was the country with the most papers (36%). Two articles did not report funding entities, while thirty reported public funding and seven reported private funding. Some studies evaluated other aspects of *C. albicans* biofilms separately from the ones of the inclusion criteria, such as adhesion capacity (31%), yeast-hyphal transition (46%) and visualization by SEM/CLSM/fluorescence microscope (44%) (Table 1).

### 2.2. Risk of Bias Assessment

All included studies were assessed for the risk of bias. Out of the thirty-nine articles, no study presented a high risk of bias, thirty articles showed low risk and nine studies had a medium risk of bias. Item 4 (presence of die control in cytotoxic activity) was the least reported (23%), while items 1 and 3 were reported by 97% and 92% of the studies, respectively. Thirty-five studies mentioned the number of replicates or repetitions of experiments, which is important to validate data. Complete scores of all items are described in Table 2, according to the parameters considered in the analysis.

### 2.3. Type of Plant Derivatives

The studies evaluated different plant derivatives: extracts from plants were the most studied derivatives (thirteen articles), followed by essential oils (six studies) and terpenes (six studies). Other compounds evaluated were alkaloids, flavonoids, rich fractions, polyphenols and naphthols.

The majority of the studies reported the anti-*C. albicans* biofilm activity and cytotoxic effect of one plant derivative (26/39 articles), while thirteen studies evaluated at least two plant derivatives; one study evaluated seven extracts of *Casearia sylvestris* [19]. Three studies evaluated at least one sample of plant extracts and other compounds of a different chemical nature, such as essential oils [16], phenols [24] and fractions [54]. Additionally, one article evaluated flavonoids and diterpenes [46].

### 2.4. Cell Types in the Research Studies

Cytotoxic activity was performed on human cells in 67% of the studies, while 33% used cells derived from other animals. Most of the studies analyzed the plant derivatives’ activity in cell lines (twenty-six studies), whilst ten studies used primary cells and three studies examined bank and primary cells.

Epidermal cells were examined by seventeen studies, and eight studies evaluated cytotoxicity on macrophages. The most used cell line was murine macrophage cells (RAW 264.7) (6/40 articles), followed by human umbilical vein endothelial cells (HUVEC) (5/40 articles), human keratinocytes cells (HaCaT) (4/40 articles) and mouse fibroblast cells (L929) (4/40 articles). In one study, the hemolytic effect on human erythrocytes from a healthy person was evaluated [25].

Two studies [29,42], those of Ma et al. (2015) and Oliveira et al. (2017), analyzed the effect of plant derivatives on the viability of four different cell types, but most studies (28/39) evaluated the cytotoxicity over one cell line (Table 3).

### 2.5. C. albicans Strain

We can identify the use of thirteen different *C. albicans* reference strains in the thirty-nine studies included in this review. All studies examined at least one reference strain, the most used ones being SC 5314 (eleven studies), ATCC 10,231 (twelve studies), ATCC 90,028 (six studies) and ATCC 18,804 (four studies). Six studies used two or more strains, and four studies always jointly included clinical isolates with one or more reference strains (Table 4).

Sharma et al. (2019) and Kim et al. (2017) [39,44] included an experimental design with a sensitive and fluconazole-resistant strain. In the study of Souza et al. (2018) [28], nine clinical strains from kidney transplant patients were included, whereas the study of Sudjana et al. (2012) [37] included seven clinical isolates without data on the characteristics of the patients from which they were obtained. Ivanov et al. (2021) [51] used three clinical isolates from the oral cavities of patients from the Clinical Hospital Center Zvezdara (ENT clinic.), and Curvelo et al. (2014) [38] used one clinical strain from the oral mucosa of human-immunodeficiency-virus-positive pediatric patients resistant to fluconazole (Table 4).

### 2.6. Cell Cytotoxicity

Various assays were used to determine the cytotoxicity of plant derivatives. A total of 72% of the included articles used the tetrazolium assay (MTT), followed by Sulforhodamine B assay (1%). Other evaluation techniques consisted of a cytotoxicity detection kit, neutral red dye method, resazurin and Alamar blue^®^.

Different schemes of exposure times to plant derivatives were used, from 1 min to 72 h. A 24 h exposure was the most used (21/39), while nine studies used shorter exposure times, between 5 min and 2 h.

According to international standards (DIN EN ISO 10993-5:2009) guidelines for the cytotoxicity classification, plant derivatives are not cytotoxic when the inhibition of cell viability is less than 25%; slightly cytotoxic if inhibition is between 25 and 50%; moderately cytotoxic with inhibition between 50 and 75%; and strongly cytotoxic with inhibition higher than 75%, in comparison to the control group. According to this, plant derivatives in twelve studies did not display cytotoxic effects, two studies found slight toxicity and fourteen studies reported moderate and strong cytotoxicity.

Data from some studies did not allow researchers to classify cytotoxicity because the results were presented as CC50, IC50 or GI50. Moreover, three studies did not report values of CC50, IC50 or GI50 at the highest concentrations evaluated, and two studies indicated that the compounds did not display cytotoxicity, but it was not possible to extract information on the percentage of cellular viability after treatment.

In the study by de Oliveira et al. (2017) [42], four cell lines were treated with *Rosmarinus officinalis* extract, obtaining moderate cytotoxic effects in three cell lines, while in human breast carcinoma cells (MCF-7), the extract did not produce toxicity. Sudjana et al. (2012) [37] evaluated the essential oil of *Melaleuca alternifolia* in short exposure times, finding no cytotoxic effect in the three cell lines used.

Six of the studies that used 24 h of exposure did not identify cytotoxicity, two studies displayed results with slight cytotoxicity and eight studies reported plant derivatives with moderate and strong toxicity. Eight studies evaluated extracts on different cell lines, of which five reported no cytotoxicity or viability percentages >50%, even at concentrations of 25,000 μg/mL, as in the case of the extract of *Arctium lappa* [41]. Three studies described a cytotoxic effect in a concentration range from 100 μg/mL to 3125 μg/mL.

Exposure times lower than two hours were used in four studies, one of which reported that the essential oil of *Melaleuca alternifolia* [37] did not present cytotoxicity in different cell lines. Three studies showed a strong cytotoxicity of the extract of *Eugenia uniflora* [28], essential oil of *Santolina impressa* [49] and Allyl isothiocyanate of cruciferous plants [25] at concentrations between 200 μg/mL and 1050 μg/mL. Similarly, a study that evaluated seven extracts [19] reported results of slight and moderate toxicity with short treatment times.

Cytotoxicity curves were reported in three studies [34,50,53], evaluating 2, 3 and 7 different exposure times. Two of these studies reported similar results; one study did not identify any toxicity of the compound Hinokitiol [50] in the time range between 1 and 60 min, and, in another study, a strong cytotoxicity of the extracts of *Cryptocarya mandioccana* and *Cryptocarya moschata* [53] was found after exposure times of 6, 12 and 24 h.

Two studies evaluated Hinokitiol [44,50], finding similar results without a cytotoxic effect on the cells evaluated at concentrations of 1.6 μg/mL and 0.25 mM.

The compounds magnoflorine, ellagic acid, myricetin and eucarobustol from the leaves of *Eucalyptus robusta*, flavonoids and diterpenes of *Prunus cerasoides*, flavonoids from *Moringa oleifera*, an extract of *Buchenavia tomentosa* and an extract of *Arctium lappa* [20,24,30,35,41,46,48] should be noted because they did not present cytotoxicity at concentrations greater than or equal to those that have effect on *C. albicans* biofilms. A full description of cytotoxicity results can be found in Table 3.

### 2.7. Antibiofilm Activity

Seventeen of the included studies evaluated plant derivatives in models that allow the identification of whether inhibition occurs in *C. albicans* biofilm formation (BF); six studies evaluated the inhibition capacity on preformed biofilms (PB); and, in sixteen studies, both assessments were performed.

The most used techniques for the evaluation of biofilm formation (BF) inhibition were crystal violet (sixteen studies), XTT (sixteen studies) and viable counts (CFU/mL) (five studies). Other techniques used were dry weight measurement, alamar blue assay and MTT. In eight studies, the use of two techniques for the evaluation of BF was identified.

Twenty-three articles reported evaluations in PB, with the most used techniques being XTT (eleven studies), crystal violet (seven studies) and viable count (CFU/mL) (seven studies). Other techniques used in a smaller number of articles were MTT (three studies), SEM and fluorescence microscopy.

In the case of evaluations of BF, the most used exposure times were 24 h (twenty-five studies) and 48 h (seven studies). With regard to PB, the most used combination was 24 h of formation with 24 h of exposure (twelve studies). Other schemes, used in fewer studies, were 48 h of formation with 24 h of exposure (two studies) and 24 h of formation per 1 h of exposure (two studies).

Three BF studies used two exposure times, while, for PB, one study used four exposure times, another used five and the rest of the studies used one exposure time in different formations of the biofilm times, with 24 h being the most used exposure (fourteen studies).

Out of all the studies evaluating BF, 48.5% (15/33) showed inhibition results of the evaluated plant derivatives—lower than 60%—including two studies in which the evaluated derivatives did not display any antibiofilm activity. For PB, 39.1% (9/23 studies) of studies reporting this same level of inhibition were found.

As they show percentages of inhibition greater than 90% in BF models, we call attention to the flavonoids extracted from *Moringa oleifera* [30] seed coating (0.42 mg/mL), *Cinnamomum burmannii* extract, Cinnamon bark essential oil extracted from *Cinnamomum verum* [16], nepodin from *Rumex crispus* (*Polygonaceae*) [17], extract of *Cymbopogon citratus* (0.625 mg/mL) [32], myricetin and essential oil of *Melaleuca alternifolia* [37]. Out of these, in the PB evaluation, the flavonoids extracted from *Moringa oleifera* seed coating (0.42 mg/mL) and the extract of *Cymbopogon citratus* (0.625 mg/mL) showed reductions close to 80% with a 24 h exposure for flavonoids extracted from the *Moringa oleifera* seed coating in BF and PB, while, for *Cymbopogon citratus*, the treatment time was 8 h in PB.

Other compounds, such as dioscin [22] from herbs and vegetables of the *Dioscorea* genus (4 μg/mL) and dracorhodin perchlorate [23] from the exudates of the fruit of *Daemonorops draco* (32 μM), produced 89 and 80% inhibition in BF, respectively. In the first case (dioscin from herbs and vegetables of the *Dioscorea* genus), the evaluation in PB showed a reduction of 50% or more at 16 μg/mL, while dracorhodin perchlorate (64 μM) produced a reduction in PB of 20%, with treatments of 24 h.

Four studies included clinical isolates in their evaluations, of which three used BF evaluation and one used BF and PB evaluations. Three of these works identified that extracts from *Eugenia* spp. [28], essential oils extracted from *Melaleuca alternifolia* [37] and β-citronellol [39] displayed an inhibitory effect greater than 60% in the formation of biofilms of clinical *C. albicans.*

The concentrations at which these derivatives reach high percentages of inhibition are highly variable and, in some cases, range from micrograms to milligrams. The use of different measurement units was observed: molarity, *p*/*v* and *v*/*v*, used according to the nature of the evaluated derivative. A full description of plant derivatives’ activity can be found in Table 4.

## 3. Discussion

The increasing *C. albicans* antifungal resistance, partially attributed to its ability to form biofilms, has led to the search for therapeutic alternatives in plant derivatives, especially aiming to produce less cytotoxic effects.

This scoping review provided a good quality overview of in vitro studies on *C. albicans* biofilms and cytotoxic effects of plant derivatives, and included thirty-nine studies, thirty of which showed a low bias risk, ensuring an adequate quality, but also demonstrated some common limitations. Thirty studies did not clearly identify the death controls of the cytotoxic study techniques, eight studies did not provide completely defined outcome measures, seven studies did not clearly identify standard methods used to evaluate activity on planktonic cultures, six studies did not mention the used solvent to prepare the plant derivatives, five studies did not describe the process to define the concentrations of plant derivatives to be evaluated, four studies did not provide the number of replicates and repetitions of each test, four studies did not describe the statistical methods and three studies did not mention the control in the biofilm activity assessment.

A country with great biodiversity such as Brazil was expected to be the largest producer of this type of study (36%) and it was also expected that the public sector would show interest in financing them (twenty-nine studies), due to the possibility of developing bioeconomies that can be facilitated by the results.

Some studies evaluated other aspects of *C. albicans* that were not considered as inclusion criteria, but that complemented the knowledge of the plant derivatives’ effects. This is the case of the studies that evaluated inhibitory activity at the planktonic level and compared the results with activity in sessile cells, as in the studies by Yang et al. (2018) and Yang et al. (2018) [22,23], where they reported the inhibition of the adhesion of *C. albicans* to polystyrene surfaces in a dose-dependent manner by dioscin and dracorhodin perchlorate. In other studies, the morphosis of *C. albicans* was evaluated by derivatives such as Allyl Isothiocyanate, Sanguinarine and β-citronellol [25,39,40], which showed an effect in the yeast to hyphal transition and had effects on the structure of the biofilm through visualization by SEM/CLSM/fluorescence microscope (Table 1).

The literature analysis highlighted great variability among the selected studies, particularly regarding the cell type investigated, including twenty-six cell lines and ten primary cells. Variability also concerned *C. albicans* strains (thirteen reference strains were identified), the assays performed and the exposure conditions applied. Preparations from twenty-three plants demonstrated activity equal to or higher than 50% against *C. albicans* biofilms in BF or PB, seven of which were essential oils and eight were extracts. Some of the plant families were *Myrtaceae* (*Eucalyptus robusta*, *Melaleuca alternifolia*, *Eugenia uniflora*, *Eugenia leitonii*, *Eugenia brasiliensis*), *Asteraceae* (*Santolina impressa*, *Artemisia judaica*), *Arecaceae* (*Daemonorops draco*), *Campanulaceae* (*Adenophora triphylla*), *Combretaceae* (*Buchenavia tomentosa*), *Dioscoreaceae* (*Dioscorea* genus), *Lamiaceae* (*Vitex gardneriana*), *Lauraceae* (*Cinnamomum burmannii*, *Cinnamomum verum*, *Cryptocarya mandioccana*, *Cryptocarya moschatta*), *Lythraceae* (*Punica granatum sarcotesta*), *Magnoliaceae* (*Magnolia officinalis*), *Menispermaceae* (*Fibraurea recisa*), *Piperaceae* (*Piper claussenianum*), *Poaceae* (*Cymbopogon citratus*) and *Papaveraceae.*

We found that the in vitro cytotoxic effect was determined by different techniques, including different schemes of exposure times. Since 24 h of exposure was the most used scheme, the little variability allowed comparisons to be made. However, it is important to consider that each study adapted the methodology according to the possibility of clinical application expected for the derivative under study. Additionally, shorter exposure times naturally generate less cytotoxicity, as exemplified in the study of Nakamura et al. (2010) [50], in which hinokitiol did not present cytotoxicity in the time range between 1 and 60 min, while Oliveira et al. (2021) [53] evaluated *Cryptocarya* spp. extracts’ cytotoxicity at exposure times of 6, 12 and 24 h, and found strong toxicity at all times.

According to the analyzed publications, the predominant methodology was metabolic activity assays, whose results were expressed as CC50, CE50, CL50, IC50, DL50, GI50 and percentage cell viability. The difficulty in comparing the results between studies was one limitation due to the lack of consensus, since several factors, such as the origin and chemical composition of the oils, extracts and others, and particularly the selected cell lines, the technical conditions and the solvent used to dilute the plant derivatives, can influence the results of the in vitro tests [55].

As per the ISO 10993-5:2009 guidelines for cytotoxicity classification, the plant derivatives of twelve studies presented neither a cytotoxic effect nor slight toxicity, which is a key characteristic to consider along with the possible antifungal capacity. However, the derivatives of the fourteen studies showing moderate and strong cytotoxicity may have many other pharmacological properties, which label this as a future potential agent to treat many diseases, including cancer.

In the study by de Oliveira et al. (2017) [42], four cell lines were treated with *Rosmarinus officinalis* extract, obtaining moderate cytotoxic effects in three cell lines, while, in human breast carcinoma cells, MCF-7, the extract did not produce toxicity. Sudjana et al. (2012) [37] evaluated the essential oil of *Melaleuca alternifolia* at short exposure times. These findings reinforce the need to include different cell lines in studies and to establish cytotoxicity evaluation models consistent with the intended clinical applications.

Two of the included studies evaluated hinokitiol with similar results; no cytotoxic effect on the cells evaluated at the concentrations of 1.6 μg/mL and 0.25 mM. It is noteworthy that the evaluation methodologies used were different; Kim et al. (2017) [44] evaluated human-bone-marrow-derived mesenchymal stem cells, MSC, at prolonged exposure times (48 h) using the MTT assay, while Nakamura et al. (2010) [50] used epithelial cells, Ca9–22, in short exposure times (1 to 60 min) and a cytotoxicity detection kit.

According to the European Committee on Antimicrobial Susceptibility Testing (EUCAST), the antifungal clinical breakpoints are between 0.001 mg/L and 16 mg/L [56]. Using EUCAST guidelines in this review, the most active derivatives in planktonic culture were dioscin (4 μg/mL) [22], *Eugenia leitonii* extracts (15.62 μg/mL) [31], *Eugenia brasiliensis* extracts from seeds (15.62 μg/mL) [31], eucarobustol E [35] (16 μg/mL)./mL), licochalcone A [36] (6.25–12.5 μg/mL), glabridin [36] (12.5–6.25 μg/mL), sanguinarine [40] (3.2 μg/mL), pomegranate extract from *Punica granatum* [43] (3.9 μg/mL) and hinokitiol [50] (1.6 μg/mL). Although EUCAST does not have antifungal breakpoints for biofilms, if we compare the current breakpoints with the results on biofilms, the following plant derivatives could be considered to inhibit (≥50%) *C. albicans* biofilm either in BF or in PB: dioscin [22] (4–16 μg/mL), *Adenophora triphylla* var. *japonica* extract [52] (6.25 μg/mL), chitin-binding lectin (PgTeL) from *Punica granatum* sarcotesta [21] (0.39 μg/mL), magnolol and honokiol from *Magnolia officinalis* [27] (16 μg/mL), roemerine from *Fibraurea recisa* [29] (8 μg/mL), sanguinarine [40] (1.6–3.2 μg/mL), nepodin from *Rumex crispus* (2–5 μg/mL), *Rumex japonicus* extract (5 μg/mL) [17], licochalcone A (2 μg/mL) and hinokitiol (3.1 μg/mL) [36,44]. Among these, dioscin, sanguinarine and hinokitiol presented an inhibitory effect on planktonic cells and both biofilm models, with results expressed in *p*/*v*.

Since comparing the results in different concentration units is not possible, it is difficult to identify the derivatives with the best inhibitory characteristics, and it would be desirable to reach a consensus for future studies. Nevertheless, it is possible to identify the following as the plant derivatives displaying percentages of inhibition higher than 90% in BF models of *C. albicans* biofilms, regardless of their concentration or the strain evaluated: flavonoids from *Moringa oleifera* seed coating [30], *Cinnamomum burmannii* extract, cinnamon bark essential oil extracted from *Cinnamomum verum*, nepodin from *Rumex crispus* (*Polygonaceae*), extract of *Cymbopogon citratus* (0.625 mg/mL), myricetin and essential oil of *Melaleuca alternifolia* [16,32,37] (Table 4). These preparations included essential oils, extracts, naphthols and flavonoids. Most of the plant preparations acted on biofilm formation or mature biofilms above 50%, turning several of these derivatives into promising options for the control of *C. albicans* biofilms; for example, in abiotic surfaces.

Antibiofilm activity may vary between plants, even in the same family. Although few of the studies included in this review used the same plant derivatives, this can be observed in the case of the studies carried out with the plant derivatives of the *Myrtaceae* family [28,31,35,37], where there are differences in the inhibitory activity on *C. albicans* biofilms, perhaps due to the chemical nature of the derivatives, the genus of the plants or the different *C. albicans* strain used. Similarly, three or four studies reported that the derivatives did not present cytotoxic effects on the viability of animal cells.

Even when flavonoids extracted from the *Moringa oleifera* seed coating (0.42 mg/mL) and the extract of *Cymbopogon citratus* (0.625 mg/mL) [30,32] showed the best reductions (80%) in PB, taking into account the set of results of cytotoxicity and the inhibitory effect on *C. albicans* biofilms, it can be said that the following compounds have a special potential: magnoflorine, ellagic acid, roemerine, myricetin, camphor, licochalcone A, nepodin from *Rumex crispus* (*Polygonaceae*), eucarobustol from the leaves of *Eucalyptus robusta*, flavonoids and diterpenes of *Prunus cerasoides*, flavonoids from *Moringa oleifera*, Sanguinarine from *Papaveraceae* family, chitin-binding lectin (PgTeL) from *P. granatum sarcotesta*, cinnamon bark essential oil extracted from *Cinnamomum verum*, essential oil of *Santolina impressa*, extract of *Rosmarinus officinalis*, extract of *Eugenia uniflora*, *Adenophora triphylla* var. *japonica* extract, *Cinnamomum burmanni* extract, extract of *Buchenavia tomentosa*, extract of *Punica granatum* and extracts from *Eugenia leitonii* (seed) and *Eugenia brasiliensis* (seed and leaf). This conclusion is reached because they did not present cytotoxicity at concentrations higher than or equal to those that had an inhibitory effect on *C. albicans* biofilm.

## 4. Limitations of the Study

In this review, the investigation was limited to in vitro cell populations, both for cytotoxicity and inhibition *C. albicans* biofilm, with the aim of facilitating the selection of derivatives to study in the future and given the great variety reported in the literature.

As mentioned before, the numerous variables related to the technical aspects of obtaining plant derivatives and their subsequent preparation, toxicity evaluation and anti-biofilm capacity, as well as the variety of valid ways to express the results, constitute the main limitation for the analysis of the literature.

## 5. Conclusions

The results of this review show that, among a variety of plant derivatives that have been studied for their inhibitory effect on *C. albicans* biofilms and for their cytotoxic capacity, several can be considered as promising for the development of future research and bioproducts applicable to human health. Those capable of generating biofilm inhibition with short exposure times, as in the case of extract of *Cymbopogon citratus*, and those that combine an anti-biofilm effect with short exposure (<8 h) and a low cytotoxic effect, such as *Buchenavia tomentosa* extract, *Rosmarinus Officinalis* extract, cinnamon bark essential oil extracted from *Cinnamomum verum* and flavonoids extracted from *Moringa oleifera* seed coating, are particularly interesting for future studies in experimental models, including controlled clinical trials. In order to move forward in the study of these and other plant derivatives with promising results, it is important to consider the need for consensus when conducting in vitro experiments and reporting their results to produce comparable information.

## 6. Materials and Methods

This scoping review was conducted according to the Preferred Reporting Items for Systematic Reviews and Meta-Analyses (PRISMA) for Scoping Reviews. The aim was to identify the current state of in vitro research on the ability of plant derivatives to inhibit *C. albicans* biofilms, considering their possible cytotoxic effect on mammalian cells. The research team constructed the research question in accordance with the Population, Concept and Context (PCC) policy format [57] (Table 5).

### 6.1. Search Strategy

Two different reviewers carried out the search process. Specific search strategies were developed and implemented using the following electronic databases: Science Direct, PubMed, Scopus and Lilacs, which were limited to 28 February 2022. The observation period was of approximately twelve years, set between 2010 and 2022. A search strategy was developed using MeSH terms, and adjustments were made to match the same terms in different search engines across the four databases (Table 6), combined with database-specific filters.

### 6.2. Criteria for the Eligibility of the Studies

Two authors assessed all papers. Eligibility criteria were defined based on the PCC for institutional methodology for scoping reviews. The following study criteria were considered for inclusion: (i) articles assessing the effects of natural compounds from plants on *C. albicans* anti-biofilm activity; (ii) articles providing a full description of the methods and results; (iii) articles describing the antifungal activity of single or combined compounds, as long as they were of natural origin; (iv) articles reporting cytotoxic activity assessed by in vitro experimental methods; and (v) articles in English and Spanish. Reviews, books, chapters and studies evaluating cytotoxicity in vivo and in situ were excluded.

### 6.3. Risk of Bias Assessment

Researchers assessed the risk of bias from individual studies. The assessment was adapted from previous systematic reviews [58]. We used nine parameters to evaluate the quality of each study: (1) description of the method for plant derivatives obtention, (2) solvent used, (3) mention of a control in the biofilm activity assessment, (4) the presence of a control in the cytotoxic activity assessment, (5) description of the number of replicates and repetitions of each test, (6) use of standard methods for activity determination on planktonic cultures, (7) description of the process to define the concentrations of plant derivatives to be evaluated, (8) description of statistical methods used, and (9) completely defined measures of outcome. Publications reporting fewer than four items were classified as having a high risk of bias, whereas those reporting more than six were classified as low risk.

### 6.4. Data Extraction Process, Synthesis and Analysis

All article titles initially found in the search were selected based on the eligibility criteria and duplicates were eliminated. Titles were read, and those that did not indicate relevance were excluded. The inclusion criteria for the abstract-based selection stage were in vitro studies that investigated plant derivatives’ inhibition of *C. albicans* biofilms.

The reviewers read the full texts of potentially eligible studies based on the inclusion/exclusion criteria, and any disagreements were resolved in consultation with another author. Only papers with all the eligibility criteria were included.

Scientific and technical information were extracted using a data table in Microsoft^®^ Office Excel^®^ (Version 2211, Redmond, WA, USA). The following data were tabulated, from the qualitative analyses, about what is currently known in the literature regarding the activity of plant derivatives in *C. albicans* biofilms and human cells: author(s), year of publication, strain, compound evaluated, solvent (for plant derivatives’ preparation), type of plant derivative (such as extract, essential oil, terpene and protein), planktonic methodology and results, evaluation technique on biofilm, time exposure in biofilm, result on biofilm formation or preformed biofilm, cell lines or animal cells evaluated and cytotoxicity technique and results.

## Figures and Tables

**Table 1:** Additional study variables to those included in the inclusion criteria and funding sources.

Reference	Study	MIC	Adhesion Test *	Yeast-Hyphal Transition Test	SEM/CLSM/Fluorescence Microscope	Funding
[16]	(Veilleux and Grenier, 2019)		X			Laboratoire de Contrôle Microbiologique de l’Université Laval
[17]	(Lee et al., 2019)	X		X	X	Basic Science Research Program and Priority Research Centers Program by the Ministry of Education of Republic of Korea
[18]	(Yang et al., 2019)	X	X	X	X	Natural Science Foundation and Financial Foundation for Medicine of Jilin Province, and National Natural Science Foundation of China
[19]	(Ribeiro et al., 2019)	X				São Paulo Research Foundation and scholarships plus overhead funds. The National Council for Scientific and Technological Development in association with FAPESP
[20]	(Kim et al., 2018)	X				Basic Science Research Program through grants from the National Research Foundation of Korea (NRF) funded by the Ministry of Education and IPET
[21]	(da Silva et al., 2018)	X				Coordenação de Aperfeiçoamento de Pessoal de Nível Superior and the Fundação de Amparo à Ciência e Tecnologia do Estado de Pernambuco
[22]	(Yang et al., 2018)	X	X	X	X	Natural Science Foundation of Jilin Province
[23]	(Yang et al., 2018)	X	X	X	X	Natural Science Foundation of Jilin Provice and the National Natural Science Foundation of China
[24]	(Lourenção Brighenti et al., 2017)	X				São Paulo Research Foundation (FAPESP) and by PROPe-UNESP/FUNDUNESP
[25]	(Raut et al., 2017)	X	X	X	X	ΜGC, New Delhi, award of Dr. D.S. Kothari Postdoctoral Fellowship
[26]	(Sadowska et al., 2017)	X	X	X		The National Science Center, Poland
[27]	(Sun, Liao, and Wang, 2015)	X	X	X	X	National Natural Science Foundation of China, Jiangsu Province Natural Science Foundation, Doctoral Fund of Ministry of Education of China, China Postdoctoral Science Foundation and Fundamental Research Funds of Southeast University
[28]	(Souza et al., 2018)	X	X			Conselho Nacional de Desenvolvimento Científico e Tecnológico and the Coordenação de Aperfeiçoamento de Pessoal de Nível Superior
[29]	(Ma et al., 2015)	X		X	X	Natural Science Foundation of China, the China National 973 Program and the Science Foundation of Yunnan Province
[30]	(Onsare and Arora, 2015)	X				University Grants Commission, India, to Guru Nanak Dev University, Amritsar. The scholarship by the Government of India through Indian Council for Cultural Relations and support of the Government of Kenya
[31]	(Sardi et al., 2017)	X			X	National Council for Scientific and Technological Development São Paulo Research Foundation, Brazil
[32]	(Madeira et al., 2016)	X			X	Foundation for Research and Scientific Development of Maranhão
[33]	(Abu-Darwish et al., 2016)	X		X		Fundação para a Ciência e a Tecnologia, Portugal
[34]	(Sadowska et al., 2014)	X	X	X		National Science Centre, Poland
[35]	(Rui-Huan et al., 2017)	X		X	X	National Natural Science Foundation of China, the Program for Changjiang Scholars and the Innovative Research Team in University, the Priority Academic Program Development of Jiangsu Higher Education Institutions (PAPD), and Fundamental Research Funds for the Central Universities
[36]	(Messier and Grenier, 2011)	X		X		The Fondation de l’Ordre des Dentistes du Québec
[37]	(Sudjana et al., 2012)	X	X			Rural Industries Research and Development Corporation, Australia
[38]	(Curvelo et al., 2014)	X		X		Conselho Nacional de Desenvolvimento Científico e Tecnológico, by the Fundação Carlos Chagas de Amparo à Pesquisa do Estado do Rio de Janeiro and by the Coordenação de Aperfeiçoamento de Pessoal de Nível Superior
[39]	(Sharma et al., 2020)	X		X	X	Indian Council for Medical Research, Government of India for awarding senior research fellowship
[40]	(Zhong et al., 2017)	X		X	X	National Natural Science Foundation of China, the National Key Basic Research Program of China, and the Shanghai Pujiang Program
[41]	(de Oliveira et al., 2014)	X				None
[42]	(de Oliveira et al., 2017)	X				None
[43]	(Endo et al., 2012)	X				Conselho Nacional de Desenvolvimento Científico e Tecnológico, Financiadora de Estudos e Projetos, Capacitação e Aperfeiçoamento de Pessoal de Nível Superior, Fundação Araucária, and Programa de Pós-graduação em Ciências Farmacêuticas da Universidade Estadual de Maringá
[44]	(Kim et al., 2017)	X			X	Korea University
[45]	(Vale et al., 2019)	X			X	PQ-BPI/FUNCAP, Brazil
[46]	(Arora and Mahajan, 2019)	X				ΜGC New Delhi University with Potential for Excellence (UPE) Scholarship
[47]	(Rivas da Silva et al., 2012)	X				Coordenação de Aperfeiçoamento de Pessoal de Nível Superior (CAPES), Conselho Nacional de Desenvolvimento Científico e Tecnológico (CNPq) and Fundação de Amparo a Pesquisa do Estado do Rio de Janeiro (FAPERJ)
[48]	(Mo et al., 2020)	X			X	Shanxi National Science Foundation, Fundamental Research Funds for the Central Universities and the Project of Independent Innovative Experiment for Postgraduates in medicine in Xi’an Jiaotong University
[49]	(Alves-Silva et al., 2019)	X		X		Fundação para a Ciência e a Tecnologia and Centro 2020 Regional Operational Program, Portugal
[50]	(Nakamura et al., 2010)		X			Development of Scientific Research from the Ministry of Education, Science, and Culture of Japan and the Ministry of Health, Labor and Welfare
[51]	(Ivanov et al., 2021)	X		X		Serbian Ministry of Education, Science and Technological Development
[52]	(Kim and Kim, 2021)	X	X	X		GRRC Program of Gyeonggi province, Republic of Korea
[53]	(de Oliveira Zoccolotti et al., 2021)	X			X	Coordenação de Aperfeiçoamento de Pessoal de Nível Superior-Brasil (CAPES)
[54]	(Pereira et al., 2016)	X			X	São Paulo Research Foundation (SP, Brazil); National Council for Scientific and Technological Development (CNPq, Brazil); and a partnership between the CNPq and the Coordination for the Improvement of Higher Education Personnel (CAPES, Brazil)

* Studies included adhesion tests on abiotic and biotic surfaces (cell cultures).

**Table 2:** Risk of bias assessment.

Reference	Study	Item									Score	Bias Risk
		1	2	3	4	5	6	7	8	9		
[16]	(Veilleux and Grenier, 2019)	X	X	X		X	X	X	X	X	8	Low
[17]	(Lee et al., 2019)	X	X	X		X	X		X	X	7	Low
[18]	(Yang et al., 2019)	X	X	X		X	X	X	X	X	8	Low
[19]	(Ribeiro et al., 2019)	X	X	X	X	X		X	X		7	Low
[20]	(Kim et al., 2018)	X	X	X		X	X	X	X	X	8	Low
[21]	(da Silva et al., 2018)	X	X	X		X		X	X		6	Medium
[22]	(Yang et al., 2018)	X	X	X		X	X	X	X	X	8	Low
[23]	(Yang et al., 2018)	X	X	X		X	X	X	X	X	8	Low
[24]	(Lourenção Brighenti et al., 2017)	X	X	X	X	X	X	X	X	X	9	Low
[25]	(Raut et al., 2017)	X	X	X	X	X	X	X	X	X	9	Low
[26]	(Sadowska et al., 2017)	X	X	X	X	X	X	X	X	X	9	Low
[27]	(Sun, Liao and Wang, 2015)	X	X	X		X	X	X	X	X	8	Low
[28]	(Souza et al., 2018)	X		X	X	X	X	X	X		8	Low
[29]	(Ma et al., 2015)	X	X	X			X			X	5	Medium
[30]	(Onsare and Arora, 2015)	X	X	X	X	X		X	X	X	8	Low
[31]	(Sardi et al., 2017)	X	X	X		X	X	X	X	X	8	Low
[32]	(Madeira et al., 2016)	X	X	X		X	X	X	X		7	Low
[33]	(Abu-Darwish et al., 2016)	X	X	X	X	X	X	X	X		8	Low
[34]	(Sadowska et al., 2014)	X	X	X		X		X		X	6	Medium
[35]	(Rui-Huan et al., 2017)		X	X		X	X	X		X	6	Medium
[36]	(Messier and Grenier, 2011)	X	X	X		X	X		X	X	7	Low
[37]	(Sudjana et al., 2012)	X	X	X		X	X	X	X	X	8	Low
[38]	(Curvelo et al., 2014)	X				X	X	X	X	X	6	Medium
[39]	(Sharma et al., 2020)	X	X	X		X	X	X	X	X	8	Low
[40]	(Zhong et al., 2017)	X	X	X			X	X	X	X	7	Low
[41]	(de Oliveira et al., 2014)	X	X	X		X	X	X	X	X	8	Low
[42]	(de Oliveira et al., 2017)	X	X	X			X		X	X	6	Medium
[43]	(Endo et al., 2012)	X				X	X	X		X	5	Medium
[44]	(Kim et al., 2017)	X	X	X		X	X	X	X	X	8	Low
[45]	(Vale et al., 2019)	X	X				X	X	X		5	Medium
[46]	(Arora and Mahajan, 2019)	X	X	X		X			X	X	6	Medium
[47]	(Rivas da Silva et al., 2012)	X		X		X	X	X	X	X	7	Low
[48]	(Mo et al., 2020)	X	X	X		X	X	X	X	X	8	Low
[49]	(Alves-Silva et al., 2019)	X	X	X		X	X	X	X		7	Low
[50]	(Nakamura et al., 2010)	X		X	X	X		X	X	X	7	Low
[51]	(Ivanov et al., 2021)	X		X	X	X	X	X	X	X	8	Low
[52]	(Kim and Kim, 2021)	X	X	X		X		X	X	X	7	Low
[53]	(de Oliveira Zoccolotti et al., 2021)	X	X	X		X	X	X	X	X	8	Low
[54]	(Pereira et al., 2016)	X	X	X		X	X	X	X		7	Low

(1) Description of the method for plant derivative obtention, (2) solvent used, (3) mention of a control in the biofilm activity assessment, (4) the presence of a control in cytotoxic activity assessment, (5) description of the number of replicates and repetitions of each test, (6) use of standard methods for activity determination on planktonic cultures, (7) description of the process to define the concentrations of plant derivatives to be evaluated, (8) description of statistical methods used, and (9) completely defined measures of outcome.

**Table 3:** Principal findings of the cytotoxic effect of plant derivatives.

Reference	Study	Cell Type	Primary Cell or Cell Line	Assay Used	Plant Derivatives	Solvent Used for Plant Derivatives’ Preparation	Time Points	Principal Finding
[16]	(Veilleux and Grenier, 2019)	Human epithelial oral cells B11 Human epithelial oral GMSM-K	Cell line	MTT assay	*-Cinnamomum burmannii* extract -Cinnamon bark essential oil extracted from *Cinnamomum verum*	DMSO	24 h	Extract was not cytotoxic at 1000 µg/mL in B11 cells Extract at 500 µg/mL reduced cell viability in 42.8% in GMSM-K cells Essential oil at 0.125% reduced cell viability in 100% and 80% and in B11 and GMSM-K cells, respectively
[17]	(Lee et al., 2019)	Mouse melanocytes B16 (ATCC CRL-6322)	Cell line	MTT assay	-Nepodin from *Rumex crispus* (Polygonaceae)	DMSO	24 h	Cell viability was >50% in the presence of 200 μg/mL of nepodin
[18]	(Yang et al., 2019)	Human umbilical vein endothelial cells, HUVEC Chang liver cells	Cell line Cell line	MTT assay	Lycorine hydrochloride isolated from *Lycoris radiata*	DMSO	24 h	HUVEC cells: IC50 > 256 μM of lycorine hydrochloride Chang liver cells: IC50 > 256 μM lycorine hydrochloride
[19]	(Ribeiro et al., 2019)	Normal oral keratinocytes spontaneously immortalized, NOKsi	Cell line	MTT assay	Extracts of *Casearia sylvestris*	84.15% Ethanol and 15% DMSO	1 h	5 extracts at 500 μg/mL produced cellular inhibition between 25 and 50% and 2 extracts produced inhibition between 50 and 75%
[20]	(Kim et al., 2018)	Human keratinocytes cells, HaCaT	Cell line	MTT assay	Magnoflorine	DMSO	48 h	Magnoflorine was not cytotoxic at 600 µM
[21]	(da Silva et al., 2018)	Human peripheral blood mononuclear cell, PBMC	Primary	MTT assay	Chitin-binding lectin (PgTeL) from *Punica granatum sarcotesta*	H_2_O	72 h	Cell viability higher than 90% at 100 μg/mL of PgTeL
[22]	(Yang et al., 2018)	Human epithelial cells, JEG-3	Cell line	MTT assay	Dioscin from herbs and vegetables of *Dioscorea* genus	DMSO	24 h	IC50 of 13 μg/mL
[23]	(Yang et al., 2018)	Human umbilical vein endothelial cells, HUVEC Human placental cells, JEG-3	Cell line	MTT assay	Dracorhodin perchlorate of the exudates of the fruit of *Daemonorops draco*	DMSO	24 h	HUVEC cells: IC50 of 75.63 μM JEG-3 cells: IC50 of 65.72 μM
[24]	(Lourenção Brighenti et al., 2017)	Human epithelial oral cell, KB CCL-17 Normal kidney epithelial cells (Vero cells)	Cell line	MTT assay	Extract of *Buchenavia tomentosa* Ethanol ellagic acid	DMSO	24 h	The extract was not cytotoxic at 1600 µg/mL in KB CCL-17 cells Cell viability of Vero cells was higher than 80% in 50 µg/mL of ellagic acid
[25]	(Raut et al., 2017)	Human erythrocytes from a healthy person	Primary	Percentage of hemolysis	Allyl isothiocyanate of cruciferous plant	DMSO	1 h	90% hemolytic effect at 2 mg/mL of allyl isothiocyanate
[26]	(Sadowska et al., 2017)	Mouse fibroblast cells L929	Cell line	MTT assay	Extract from *Hippophae rhamnoides* twigs and leaves	DMSO	24 h	Twig extract: IC50 of 664.8 μg/mL Leaf extract: IC50 of 1060.4 μg/mL
[27]	(Sun, Liao and Wang, 2015)	Rat hepatic stellate cells, HSC-T6	Cell line	CCK-8 assay	Magnolol and Honokiol from *Magnolia officinalis*	DMSO	24 h	Compounds were not cytotoxic at 32 µg/mL in HSC-T6 cells
[28]	(Souza et al., 2018)	Human buccal epithelial cells	Primary	Viable cells count with trypan blue	Extract of *Eugenia uniflora*	H_2_O	1 h	Cell viability was 80% in 2000 μg/mL of extract
[29]	(Ma et al., 2015)	Human gastric cancer cells, SGC-7901 Human colon cancer cells, HT-29 Human gastric cancer cells, MGC-803 Human umbilical endothelial vein cells	Cell line Cell line Cell line Cell line	MTT assay	Roemerine from the fresh rattan stem of *Fibraurea recisa*	DMSO	24 h	SGC-7901: IC50: 0.844 mg/L of roemerine HT-9: IC50: 1.279 mg/L Of roemerine MGC-083: 0.631 mg/L (2.26 µM) of roemerine Human umbilical vein endothelial cell: IC50 of 43.047 mg/L of roemerine
[30]	(Onsare and Arora, 2015)	Sheep blood	Primary	MTT assay	Flavonoids extracted from *Moringa oleifera* seed coating	DMSO	24 h	Flavonoids were not cytotoxic to 0.42 mg/mL
[31]	(Sardi et al., 2017)	Murine macrophages cells, RAW 264.7	Cell line	MTT assay	Extracts from *Eugenia leitonii* (seed) and *Eugenia brasiliensis* (seed and leaf)	NaCl 0.9%, *w*/*v*	24 h	The *Eugenia* spp. extracts were not cytotoxic at 400 μg/mL on RAW cells
[32]	(Madeira et al., 2016)	Peripheral blood mononuclear cells	Primary	MTT assay	Extract of *Cymbopogon citratus*	Ethanol	24 h	Cell viability was <40% with 3125 μg/mL
[33]	(Abu-Darwish et al., 2016)	Murine macrophages cells, Raw 264.7 Hepatocyte cells, HepG2	Cell line Cell line	MTT assay	Essential oil of *Artemisia judaica*	DMSO	24 h	Cell viability of RAW 264.7 and HepG2 cells was <40% at 1.25 μL/mL of essential oil
[34]	(Sadowska et al., 2014)	Mouse fibroblast cells, L929	Cell line	MTT assay	saponin-rich fractions from *Medicago sativa* (aerial parts and roots) and *Saponaria officinalis*	Ethanol 1.25%	0.5 and 24 h	Exposure to 0.5 h: LD50 was >500 μg/mL and 500 μg/mL for saponin rich fractions of *M. sativa* aerial parts respectively. LD50 was 187.5 μg/mL for Saponin rich of *M. sativa* roots. Saponin fractions of *S. officinalis* reduced cell viability > 90% to 15.6 μg/mL Exposure to 24h: LD50 = 500 μg/mL and 15.6 μg/mL for saponin fractions of *M. sativa* aerial parts *M. sativa* roots respectively. Saponin fractions of *S. officinalis* reduced cell viability > 90% at 3.9 μg/mL
[35]	(Liu et al., 2017)	Human normal liver cells, LO2	Cell line	MTT assay	Eucarobustol E (EE) from the leaves of *Eucalyptus robusta*	DMSO 0.5%	48 h	Compound was not cytotoxic at 128 µg/mL
[36]	(Messier and Grenier, 2011)	Human oral epithelial cells, GMSM-K	Cell line	MTT assay	Licochalcone A, glabridin and glycyrrhizic acid	Ethanol	2 h	Glycyrrhizic acid was not cytotoxic at 20 µg/mL Glabridin reduced cell viability at 43% in 20 µg/mL Licochalcone reduced cell viability at 58% to 20 µg/mL
[37]	(Sudjana et al., 2012)	-Buccal epithelial cells-BECs -Human adenocarcinoma epithelial cells, HeLa -Human adenocarcinomic alveolar basal epithelial cells, A549	Primary Cell line Cell line	BECs: trypan blue exclusion method HeLa and A549: uptake of propidium iodide quantified by flow cytometry	Essential oil of *Melaleuca alternifolia*	Tween 80 0.001%	BECs: 5 min HeLa and A549: 90 min	Essential oil was not cytotoxic in BECS, Hela and A549 cells at 0.031%, 0.062% and 0.062% *v*/*v*, respectively
[38]	(Curvelo et al., 2014)	Mouse fibroblast cells, L929	Cell line	Neutral red dye method	Essential oil from the leaves of *Piper claussenianum*	Unidentified	48 h	CC50: 0.5%
[39]	(Sharma et al., 2020)	Human embryonic kidney cells, HEK293	Cell line	MTT assay	β-citronellol	DMSO	24 h	The viability of HEK cells reduces to ≤70% at 2000 µg/mL of β-citronellol
[40]	(Zhong et al., 2017)	Human umbilical vein endothelial cells, HUVECs	Cell line	MTT assay	Sanguinarine from *Papaveraceae* family	DMSO	24 h	IC50: 7.8 µg/mL
[41]	(de Oliveira et al., 2014)	Murine macrophages cells, RAW 264.7	Cell line	MTT assay	Extract of *Arctium lappa*	Propylene glycol	24 h	Extract was not cytotoxic in RAW cells at 250 mg/mL
[42]	(de Oliveira et al., 2017)	Murine macrophages cells, RAW 264.7 Human gingival fibroblasts, FMM-1 Human breast carcinoma cells, MCF-7 Cervical carcinoma cells, HeLa	Cell line Primary Cell line Cell line	MTT assay	Extract of *Rosmarinus officinalis.*	Propylene glycol	5 min	RAW 264.7, FMM-1 and HeLa: 100 mg/mL resulted in cell viability reached levels lower than 50% Extract was not cytotoxic in MCF-1 cells at 100 mg/mL
[43]	(Endo et al., 2012)	Kidney epithelial normal cells, Vero	Cell line	Sulforhodamine B assay	Extract of *Punica granatum*	Unidentified	48 h	CC50: 80 µg/mL
[44]	(Kim et al., 2017)	Human bone-marrow-derived mesenchymal stem cells, MSCs	Primary	MTT assay	Hinokitiol	DMSO 0.5%	48 h	Hinokitiol was not cytotoxic in MSC cells at 1.6 µg/mL
[45]	(Vale et al., 2019)	Murine dermal fibroblasts cells, L929-CCL1™ Human keratinocytes, HaCaT (HB241™)	Cell line Cell line	MTT assay	Essential oil from leaves of *Vitex gardneriana*	Tween 20 10% (*v*/*v*)	48 h	Essential oil had cytotoxic effects at 0.03–1.25% on L929 cells Essential oil had no cytotoxic effects at 2.5% (*v*/*v*)
[46]	(Arora and Mahajan, 2019)	Sheep blood	Primary	MTT assay	Flavonoids and diterpenes of *Prunus cerasoides*	DMSO	24 h	Flavonoids and diterpenes had no cytotoxic effect in sheep blood at 136.5 mg/mL and 86.5 mg/mL, respectively
[47]	(Rivas da Silva et al., 2012)	Swiss mouse peritoneal macrophages	Primary	MTT assay	(+) α-pinene ≥99% (+) β-pinene ≥98.5%	Unidentified	24 h	(+) α-pinene reduced cell viability by 100% and 66% with 0.5 mg/mL and 0.25 mg/mL, respectively (+) β-pinene reduced cell viability by 57.7% with 0.25 mg/mL
[48]	(Mo et al., 2020)	Human umbilical vein endothelial cells, HUVEC, CRL-1730	Cell line	MTT assay	Myricetin	Methanol	24 h	Compounds were not cytotoxic at 80 µg/mL
[49]	(Alves-Silva et al., 2019)	Mouse leukemic macrophage cells, RAW 264.7	Cell line	Resazurin method	Essential oil of *Santolina impressa*	DMSO (≥1%)	1 h	Cell viability <50% at 1.05 mg/mL of essential oil
[50]	(Nakamura et al., 2010)	Epithelial cells, Ca9–22	Cell line	Cytotoxicity detection kit: cell damage is detected by release of lactate dehydrogenase into the medium	Hinokitiol C10H12O2 (β–thujaplicin) of the essential oils isolated from *Cupressaceae*	Unidentified	1, 2, 5, 10, 20, 30 and 60 min	Less than 2% of cytotoxic activities of 0.25 mM for less than 30 min
[51]	(Ivanov et al., 2021)	Porcine liver primary cultured PLP2 cell	Primary	Sulforhodamine B assay	Camphor and eucalyptol	Unidentified	48 h	Camphor: GI50: >400 µg/mL Eucalyptol: GI50: 56 ± 4 µg/mL
[52]	(Kim and Kim, 2021)	HaCaT cells and human macrophages THP-1	Cell line	MTT assay	*Adenophora triphylla* var. *japonica* (Korean name Zandae) extract	DMSO 0.1%	24 h	CC50: >100 µg/mL on both cell lines
[53]	(de Oliveira Zoccolotti et al., 2021)	Normal oral keratinocytes (NOK) cells	Primary	Alamar Blue^®^ assay	Extracts of leaves and fruits of the species of *Cryptocarya mandioccana* and *Cryptocarya moschata*	PBS with ethanol 5%	6, 12 and 24 h	Extracts at 0.045 g/mL inhibits cells more than 75% at each time
[54]	(Pereira et al., 2016)	Murine macrophage, RAW264.7 Human keratinocyte, HaCaT	Cell line Cell line	MTT	Extracts and fractions from the leaves of *Sideroxylon obtusifolium* and *Syzygium cumini*	10% *v/v* ethanol	24 h	On RAW cells, *Syzygium cumini (sc)* extract was not cytotoxic at 200 µg/mL and n-butanol fraction from *S. obtusifolium* was cytotoxic to 100 μg/mL On HaCaT, the toxicity for *Syzygium cumini* (sc) extract and n-butanol (Nb) fraction from *S. obtusifolium* was 200 and 100 μg/mL, respectively.

**Table 4:** Principal findings of studies that investigated the effect of plant derivatives on *C. albicans* biofilm.

Reference	Study	*C. albicans* Strain	Biofilm Model		Assay Used	Plant Derivatives	Time Points	Principal Finding
			Formation (BF)	Preformed (PB)				
[16]	(Veilleux and Grenier, 2019)	ATCC 28366	X	X	BF: Crystal violet PB: XTT assay and residual biomass by crystal violet	*Cinnamomum burmannii.* extract. Cinnamon bark essential oil extracted from *Cinnamomum verum.*	BF: 24 h PB: 24 h formation/1 h treatment	BF: reduced 91% to 62.5 μg/mL with extract and essential oils at 0.0391% (*v*/*v*) reduced total PB: essential oils reduced biofilm viability by 48% to concentration of 0.078%(*v*/*v*)
[17]	(Lee et al., 2019)	DAY185	X		BF: crystal violet and dry weight	Nepodin from *Rumex crispus* (Polygonaceae) *Rumex japonicus* extract	BF: 24 h	BF: reduced 90–97% to 2–5 μg/mL with nepodin. Reduced 85% at 5 μg/mL of *R. japonicus* root extract
[18]	(Yang et al., 2019)	SC5314	X	X	BF: XTT assay PB: XTT assay	Lycorine hydrochloride isolated from *Lycoris radiata*	BF: 24 h PB: 24 h formation/24 h treatment	BF: viability reduced <40% to 64 μg/mL of lycorine hydrochloride PB: reduced 20–30% 16–64 μM of lycorine hydrochloride
[19]	(Ribeiro et al., 2019)	SC5314	X		BF: viable counts (UFC/mL) and crystal violet	Extracts of *Casearia sylvestris*	BF: 24 h	BF: 4 extracts showed a <50% reduction in the viable counts of the fungus to 0.50 mg/mL
[20]	(Kim et al., 2018)	KCTC7965 (ATCC10231)	X		BF: crystal violet	Magnoflorine	BF: 24 h	BF: complete inhibition at 50 μM of magnoflorine
[21]	(da Silva et al., 2018)	URM5901	X		BF: crystal violet	Chitin-binding lectin (PgTeL) from *Punica granatum sarcotesta*	BF: 24 h	BF: reduced 50% to 0.195 and 0.39 μg/mL of PgTeL
[22]	(Yang et al., 2018)	SC5314	X	X	BF: XTT assay and viable counts (UFC/mL) PB: XTT assay	Dioscin from herbs and vegetables of *Dioscorea* genus	BF: 24 h PB: 24 h formation/24 h treatment	BF: reduced 80% to 4 μg/mL PB: reduced more than 50% at 16 μg/mL
[23]	(Yang et al., 2018)	SC5314	X	X	BF: XTT assay PB: XTT assay	Dracorhodin perchlorate of the exudates of the fruit of *Daemonorops draco*	BF: 24 h PB: 24 h formation/24 h treatment	BF: reduced about 80% to 32 μM PB: reduced 20% to 64 μM
[24]	(Lourenção Brighenti et al., 2017)	ATCC18804	X	X	BF: viable counts (UFC/mL) PB: viable counts (UFC/mL)	Extract of *Buchenavia tomentosa* Ethanol ellagic acid	BF: 24 h PB: 32 h formation/5, 15, 30 and 60 min treatment	BF: 200 μg/mL, the extract was able to reduce biofilm formation PB: extract to 1600 μg/mL reduced 60–65% of viability. Elanic acid reduced 80% in 60 min at 6.4 μg/mL
[25]	(Raut et al., 2017)	ATCC90028 GMC03	X	X	BF: XTT assay and crystal violet PB: XTT assay and crystal violet	Allyl isothiocyanate of cruciferous plants	BF: 48 h PB: 24 h formation/24 h treatment	BF: reduced the viability of GMCO3 in 45–50% and 70% in ATCC90028 strain at 0.5 mg/mL of allyl isothiocyanate PB: reduced viability of 35–60% at 1 mg/mL and 90% at 2 mg/mL in both strains
[26]	(Sadowska et al., 2017)	ATCC10231	X		BF: Alamar blue assay	Extract from *Hippophae rhamnoides* twigs and leaves	BF: 24 h	BF: reduced 80,6% to ½ MIC twig extract and 15.3% by the leaf extract
[27]	(Sun, Liao, and Wang, 2015)	SC5314	X	X	BF: XTT assay PB: XTT assay	Magnolol and Honokiol from *Magnolia officinalis*	BF: 48 h PB: 24 h formation/24 h treatment	BF: reduced 50% of biofilm at 16 μg/mL of magnolol or honokiol PB: reduced 50% biofilm at 64 μg/mL of magnolol or honokiol
[28]	(Souza et al., 2018)	SC5314 9 clinical strains from kidney transplant patients	X		BF: Crystal violet and XTT assay	Extract of *Eugenia uniflora*	BF: 66 h	BF: reduced 7–79% biofilm in clinical isolates to 1000 μg/mL of extract
[29]	(Ma et al., 2015)	SC5314	X		BF: XTT assay	Roemerine from the fresh rattan stem of *Fibraurea recisa*	BF: 24 h	BF: reduced 80–90% biofilm at 8 μg/mL
[30]	(Onsare and Arora, 2015)	MTCC227	X	X	BF: Crystal violet PB: Crystal violet and XTT Assay	Flavonoids Extracted from *Moringa Oleifera* Seed Coat	BF: 24 h PB: 14 h formation/2 , 4, 6, 8 and 24 h treatments	BF: reduced the 100% of biofilm at 0.42 mg/mL. PB: reduced 76% of biofilm at 0.42 mg/mL in 24 h with a metabolic activity biofilm of 25% or lower
[31]	(Sardi et al., 2017)	ATCC90028		X	PB: viable counts (UFC/mL)	Extracts from *Eugenia leitonii* (seed) and *Eugenia brasiliensis* (seed and leaf)	PB: 48 h formation/24 h treatment	PB: all the extracts reduced 54–55% at 10MIC (156.2 μg/mL to *E. leitonni* (seed), 312.5 μg/mL to *E. brasiliensis* (seed), 156.2 µg/mL *E. brasiliensis* (leaf))
[32]	(Madeira et al., 2016)	ATCC18804	X	X	BF: viable counts (UFC/mL) PB: viable counts (UFC/mL), XTT assay, and fluorescence microscopy	Extract of *Cymbopogon citratus*	BF: 72 h PB: 72 h formation/8 h treatment	BF: reduced 90% at 1MIC (0.625 mg/mL) and >99% at 5MIC (3.12 mg/mL) PB: reduced approximately 80% of UFC of biofilm at 1MIC (0.625 mg/mL) and produced dispersion of biofilm
[33]	(Abu-Darwish et al., 2016)	ATCC10231		X	PB: Crystal violet and MTT assay	Essential oil of *Artemisia judaica*	PB: 24 h formation/24 h treatment	PB: 2.5 µL/mL of oil reduces the amount of attached biomass by more than 50%, but the biofilm displays metabolic activity higher than 50%
[34]	(Sadowska et al., 2014)	ATCC10231	X	X	BF: XTT assay PB: XTT assay	Saponin-rich fractions from *Medicago sativa* (aerial parts and roots) and *Saponaria officinalis*	BF: 24 h PB: 24 h formation/24 h treatment	BF: reduced 19.8–36.3% at 500 µg/mL of *M. sativa* (aerial parts and roots) Saponin rich fractions of *S. officinalis* extract had no effect PB: extracts reduced 16, 9, 20, and 18% at 500 µg/mL of SFs of *M. sativa* var. Radius aerial parts, roots, and *S. officinalis* root extract, respectively
[35]	(Liu et al., 2017)	SC5314	X	X	BF: XTT assay PB: Crystal violet and XTT assay	Eucarobustol from the leaves of *Eucalyptus robusta*	BF: 24 h PB: 24 h formation/24 h treatment	BF: reduced 73% at 32 µg/mL PB: reduced 92% at 128 µg/mL
[36]	(Messier and Grenier, 2011)	LAM-1 ATCC 28366	X		BF: Crystal violet	Licochalcone A.	BF: 24 h	BF: reduced 76% and 81% biofilms of LAM-1 and ATCC 28,366 strain at 2 µg/mL of licochalcone.
[37]	(Sudjana et al., 2012)	7 clinical isolates ATCC10231 ATCC90028 ATCC90029	X		BF: Crystal violet and XTT assay	Essential oil of *Melaleuca alternifolia*	BF: 24 h	BF: reduced all isolates in 69 to 100% at 0.25% *v*/*v*
[38]	(Curvelo et al., 2014)	1 clinical strain from oral mucosa of human immunodeficiency virus-positive pediatric patients, resistant to fluconazole (PRI)	X	X	BF: MTT assay PB: MTT assay	Essential oil from the leaves of *Piper claussenianum*	BF: 24 and 48 h PB: 48 h formation/48 h treatment	BF: reduced biofilm in 32.5% at 1% of essential oil in 24 h of exposure and 50.8% in 48 h PB: reduced viability in 63.9% mature biofilm at 1% of essential oil
[39]	(Sharma et al., 2020)	2 clinical strains: D-27 (FLC sensitive) and S-1 (FLC resistant) ATCC90028	X		BF: XTT assay	β-citronellol	BF: 24 h	BF: reduced all the strain 70 to 80% at MIC values (ATCC 90028: 200 μg/mL, D-27: 100 μg/mL, S-1: 250 μg/mL)
[40]	(Zhong et al., 2017)	SC5314	X	X	BF: XTT assay PB: XTT assay and SEM	Sanguinarine from *Papaveraceae* family	BF: 24 h PB: 24 h formation/24 h treatment	BF: reduced 72.9% at 1.6 µg/mL PB: reduced 68.3% at 3.2 µg/mL
[41]	(de Oliveira et al., 2014)	ATCC18804		X	PB: viable counts (UFC/mL) and SEM	Extract of *Arctium lappa*	PB: 120 h formation/5 minute treatment	PB: reduced 14 ± 4% at 250 mg/mL
[42]	(de Oliveira et al., 2017)	ATCC18804		X	PB: viable counts (UFC/mL)	Extract of *Rosmarinus officinalis*	PB: 48 h formation/5 min treatment	PB: reduced 99.9% at 200 mg/mL in 5 min
[43]	(Endo et al., 2012)	ATCC10231		X	PB: Crystal violet and MTT assay	Extract of *Punica granatum*	PB: 48 h formation/24 h treatment	PB: reduced 50% at 62.5 μg/mL by Crystal violet but metabolic activity was maintained even at 1000 μg/mL
[44]	(Kim et al., 2017)	ATCC90028 ATCC90029 KCMF20017(FLC resistant)	X	X	BF: XTT assay PB: XTT assay	Hinokitiol	BF: 24 h PB: 24 h formation/24 h treatment	BF: reduced 50% biofilm in all strains at 3.1 μg/mL PB: reduced 50% at 400 μg/mL for ATCC90028 and ATCC90029 strain and reduced 50% biofilm of KCMF20017 at 200 μg/mL
[45]	(Vale et al., 2019)	ATCC5314	X	X	BF: crystal violet and viable counts (UFC/mL) PB: crystal violet and viable counts (UFC/mL)	Essential oil from leaves of *Vitex gardneriana*	BF: 24 h PB: 24 h formation/24 h treatment	BF: reduced at least 50% biomass to 0.156% *v*/*v*; cell viability was not reduced at any concentration (>2.5% *v*/*v*) PB: reduced at least 50% biomass at 0.312% *v*/*v* and reduce around a logarithm
[46]	(Arora and Mahajan, 2019)	MTCC227	X	X	BF: crystal violet PB: crystal violet and XTT assay	Flavonoids and diterpenes of *Prunus cerasoides*	BF: 24 h PB: 24 h formation/24 h treatment	BF: reduced 48.33% at 68.25 mg/mL and 43.25 mg/mL for flavonoids and diterpenes, respectively PB: both reduced 30% of biomass and a 37% and 40% metabolic activity at 68.25 mg/mL and 43.25 mg/mL for flavonoids and diterpenes, respectively
[47]	(Rivas da Silva et al., 2012)	ATCC10231	X		BF: XTT assay	(+) α-pinene (≥99%) (+) β-pinene (≥98.5%)	BF: 48 h	BF: prevented biofilm formation in a 100% at 1MIC (3125 μg/mL) of (+) αpinene and at 2MIC (374 μg/mL) of (+) β-pinene
[48]	(Mo et al., 2020)	ATCC10231	X		BF: crystal violet	Myricetin	BF: 48 h	BF: reduced ≥98% at 80 μg/mL
[49]	(Alves-Silva et al., 2019)	ATCC10231	X		BF: crystal violet and XTT assay	Essential oil of *Santolina impressa*	BF: 24 and 48 h	BF: reduced at least the 50% of biomass and viability of biofilm at 0.07 and 0.54 mg/mL, respectively, in 24 and 48 h
[50]	(Nakamura et al., 2010)	SC5314	X		BF: XTT assay	Hinokitiol C10H12O2 (β–thujaplicin) of the essential oils isolated from *Cupressaceae*	BF: 24 h	BF: reduced 45% to 75% at 0.5 and 1.0 mM, respectively
[51]	(Ivanov et al., 2021)	Clinical isolates: 415/15, 503/15,13/15 AND reference strain: ATCC 10231	X		BF: Crystal violet	Camphor and eucalyptol	BF: 24 h	BF: Both compounds reduced the formation of biofilm biomass by more than 50% in all strains except the 13/15 strain. The inhibition in clinical isolate 13/15 was less than 40% with both compounds
[52]	(D. Kim and Kim, 2021)	ATCC 10231	X		BF: Crystal violet	*Adenophora triphylla* var. *japonica* (Korean name Zandae) extract	BF: 25 h	BF: reduced 50% at 6.25 μg/mL
[53]	(de Oliveira Zoccolotti et al., 2021)	ATCC 90028		X	PB: Alamar blue, CFU/ml	Extracts of leaves and fruits of the species of *Cryptocarya mandioccana* and *Cryptocarya moschatta*	PB: 1 h and 24 h	PB: the extracts of leaves and fruits of *C*. *mandioccana* and *C*. *moschatta* at 0.045 g/mL, and after 1 h of contact completely inhibited biofilm formation
[54]	(Pereira et al., 2016)	ATCC 10231	X		Scanning electron microscopy (SEM) and Confocal laser scanning microscopy (CLSM)	Extracts and/or fractions from the leaves of *Sideroxylon obtusifolium* and *Syzygium cumini*	BF: 72 h SEM and 48 h CLSM	BF: *n*-butanol fraction from *S. obtusifolium* severely affected the biofilm cell structures even at 62.5 μg/m. At higher concentrations (≥250 μg/mL), the damages were lethal to the cells *S. cumini* extract affected *C. albicans* biofilm cells, with deleterious effects at 125 μg/mL concentrations and evident destruction from 500 μg/mL. The fraction and the extract affected the viability of the biofilm cells when compared to the vehicle to concentrations of 312.5 μg/mL and 625 μg/mL, respectively

**Table 5:** Focused research questions presented using the PCC policy format.

PCC Element	Description
Population	Animal cells
	Biofilms of *Candida albicans*
Concept	Effect of plant derivatives
Context	In vitro research articles
	Research articles were limited to those published in English and Spanish.

**Table 6:** Search strategy used in each database.

Database	Search Terms
PubMed	((“Biofilms”[Mesh] AND “*Candida albicans*”[Mesh]) AND (“Anti-Infective Agents”[Mesh]) AND ((“Plant Extracts”[Mesh]) OR (“Antimicrobial Stewardship”[Mesh]) OR (“Antifungal Agents”[Mesh])))
Science Direct Scopus Lilacs	(((“Biofilms”) AND (“*Candida albicans*”) AND ((“Anti-Infective Agents”) AND ((“Plant Extracts”) OR (“Antimicrobial Stewardship”) OR (“Antifungal Agents”)))))

## Data Availability

Not applicable.
